# Estimating Inert Gas Bubbling from Simple SCUBA Diving Parameters

**DOI:** 10.1055/a-1342-8030

**Published:** 2021-01-27

**Authors:** Andreas Fichtner, Benedikt Brunner, Thomas Pohl, Thomas Grab, Tobias Fieback, Thea Koch

**Affiliations:** 1 Department of Anesthesiology and Intensive Care Medicine, University Hospital Carl Gustav Carus, Dresden, Germany; 2 Emergency Department, Kreiskrankenhaus Freiberg gGmbH, Freiberg, Germany; 3 Faculty of Science and Technology, University of Algarve, Faro, Portugal; 4 Scientific Diving Center, TU Bergakademie Freiberg University, Freiberg, Germany

**Keywords:** decompression, bubble grade, SCUBA diving, Doppler ultrasound, risk management

## Abstract

Inert gas bubbles frequently occur in SCUBA divers’ vascular systems, eventually leading to decompression accidents. Only in professional settings, dive profiles can be adjusted on individual basis depending on bubble grades detected through ultrasonography. A total of 342 open-circuit air dives following sports diving profiles were assessed using echocardiography. Subsequently, (Eftedal-Brubakk) bubble grades were correlated with dive and individual parameters. Post-dive cardiac bubbles were observed in 47% of all dives and bubble grades were significantly correlated with depth (r=0.46), air consumption (r=0.41), age (r=0.25), dive time (r=0.23), decompression diving (r=0.19), surface time (r=− 0.12). Eftedal-Brubakk categorical bubble grades for sports diving with compressed air can be approximated by bubble grade = (age*50 ^−1^ – surface time*150 ^−1^ +maximum depth*45 ^−1^ +air consumption*4500 ^−1^ ) ^2^ (units in years, hours, meter, and bar*liter; R ^2^ =0.31). Thus, simple dive and individual parameters allow reasonable estimation of especially relevant medium to higher bubble grades for information on relevant decompression stress after ascent. Echo bubble grade 0 is overestimated by the formula derived. However, echo might fail to detect minor bubbling only. The categorical prediction of individual decompression stress with simple bio and dive data should be evaluated further to be developed towards dive computer included automatic ex-post information for decision-making on individual safety measures.

## Introduction

Inert gas bubbles are known to cause decompression sickness in self-contained underwater breathing apparatus (SCUBA) diving accidents and occur after inert gas (specifically Nitrogen in compressed air diving) supersaturation and omitted decompression brakes and fast ascents. Inert gas saturation is not only a function of dive time, depth, and ascent speed, but also depending on individual factors like dehydration, stress, age and others 1 2 . Mainly without any symptoms, inert gas bubbles frequently occur in SCUBA divers after ascending from a dive, and the number of bubbles finally determines a symptomatic decompression incident. However, there are divers that are more prone to post-dive bubbling compared to others after the same inert gas exposure without differences in oxidative stress or antioxidant capacity 3 . A high amount of detectable bubbles in dives within normal sports diving limits is related to symptoms of decompression sickness in around 10% and even higher in mixed gas commercial diving 4 5 6 .

Said dive parameters, as well as personal health condition and exertion during the dive 7 , are contributing to bubble formation and eventually to a decompression incident. In order to avoid such, modern watch-like dive computers provide restrictive safety margins and real-time calculations of saturation and desaturation of many virtual tissue speeds during a dive but cannot entirely avoid diving accidents. Furthermore, the majority of diving accidents caused by decompression sickness is not predicted by the adopted decompression algorithm 7 .

Bubble occurrence after a dive is not stable, can be provoked by physical activity, and the typical bubble peak is 30−45 min after a dive 1 7 . Echocardiographic bubble grading as the current gold standard is non-linear, and the categorical grading somewhat obscures the potential of high diagnostic accuracy with modern ultrasound technology according to current guidelines 8 . However, since bubble occurrence is frequent and does not equal a diving accident, the diagnostic information of just medium to higher bubble grades seem to be relevant to decide on behavioral adjustments after diving.

Our aim is to find a relationship between dive profile and individual characteristics and the severity of bubble occurrence after a dive, in order to allow sports divers to estimate decompression stress as a precondition to observe appropriate safety measures, e. g., increased surface intervals and fluid intake. With this new approach, we aim to close the gap in the mainly lacking post-dive ultrasound bubble assessment to quantify the inter- and intraindividually variable decompression response and to finally help avoid diving accidents.

## Materials and Methods

We examined 41 scuba divers of different ages, gender and body characteristics in a total of 342 single and repetitive open-circuit compressed air dives within sports diving limits using wet-suit and modern real-time dive computers in shallow and deep, fresh and salt water. Dives were standard educational sports dives for research divers with underwater tasks like orientation, buoy operation, measurements, but without any heavy exercise, current or workload. Dive computer limits such as ascent speed and decompression breaks were observed and monitored by analyzing the log of the dive computers. Divers and dive profiles covered a broad spectrum and were not standardized. In contrast, post-dive physical behavior and bubble recording were standardized: All divers were assessed for weight (empty bladder) including bio-impedance estimated percentages of body fat, water and muscle content (Beurer BF 105 diagnostic scale), height, diseases, vital signs and activity level before and after any dive. Daily fluid intake was recorded throughout the whole study period for each individual in 100 ml-intervals. Surface intervals were recorded before any dive and specified in hours up to a maximum of 48 h. After dive ascent, all divers walked back to the dive base with full equipment (approx. 100 meters, provocation period), dressed off and reported directly after the dive (30 min, the time interval was recorded) for post-dive assessment without any rest, eating or drinking during this period. Dive parameters (depth, time, total air consumption, safety and decompression stops, pre-dive surface intervals up to 24 h), including impaired well-being during the dive due to stress, cold, equalization problems, and others were recorded while standing. Total air consumption was calculated using tank pressure difference (pre- and post-dive) and tank size. After urinating, body weight and impedance-derived percentages of body compositions were measured, and guided Doppler Self-Monitoring for bubble detection was performed. Forty minutes after the dives, standardized echocardiography to record inert gas bubbles was performed in laying supine position at subcostal and apical approach using a GE Logic e (GE Healthcare, Solingen) ultrasound machine with a curved array multi-frequency probe. After signal optimization and visual bubble detection within 1 min, video recordings of 30 s each were stored and later assessed again by two independent, experienced (international ultrasound diploma) and blinded sonographers. Visible bubbles were graded using the Eftedal-Brubakk-Scale 9 for visual echocardiographic assessments ( Table 1 ).

**Table:** **Table 1** Eftedal-Brubakk scale 9 for inert gas bubble grading in SCUBA divers and its approximate relation to bubble numbers in semi-automatic bubble counting 10 .

Number of bubbles per cm ^2^	Bubble Grades	Eftedal-Brubakk (EB) scale for echocardiographic bubble detection
0	BG0	No bubbles visible
0.05	BG1	Occasional bubbles
0.2	BG2	At least 1 bubble/4 cardiac cycles
1	BG3	At least 1 bubble/cardiac cycle
3.5	BG4	At least 1 bubble at every cm ^2^ in every view
10	BG5	Whiteout – no single bubble discrimination

Statistics and graphs were created using R and R Studio 4.0.2 (R Core Team, 2020, www.R-project.org ). Due to the exponential nature of the EB scale 10 , linear approximations could be used when square root transforming the EB grade for the linear regression. A big portion of the divers were measured multiple times, linear mixed effect models 11 were used to correct the repeated measures and verify the results of the linear model. We aimed at 80% power and p<0.05 to detect the difference of one grade in EB scale 12 .

Following informed consent from participants and ethical approval through the university ethics committee, as well as following the ethical standards of the International Journal of Sports Medicine 13 , the dives were monitored but not interfered with. Depending on the measurement results after their dive, the participants received safety information only. The study was supported by the German Society of Diving and Hyperbaric Medicine (GTÜM e.V.) and by General Electric Healthcare’s ultrasound division in Germany through material provisions.

## Results

### Dive and individual parameters

**Fig. 1:**
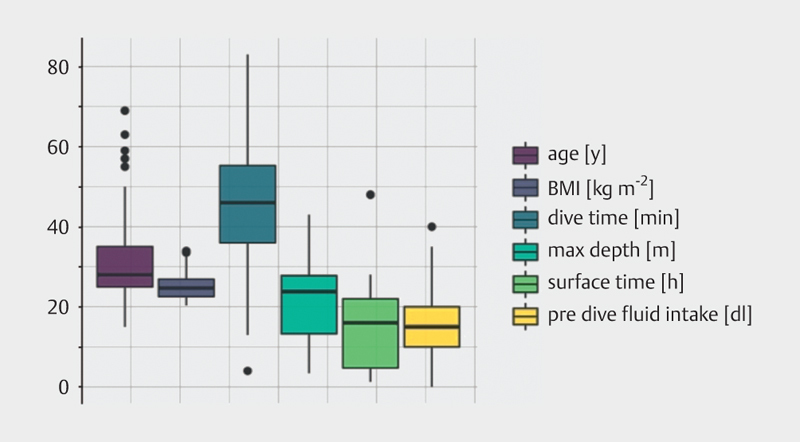
Bio-data and dive profiles of the monitored dives. Depth is always recorded as maximum depth during the dive. Dive profiles covered a broad spectrum within typical sports diving limits that were considered safe, dive computers were worn continuously, recommended safety stops and few single-step decompression stops according to commercial dive computer recommendations were followed.

Of 342 dives, 101 were completed by women and 241 by men. All divers were medically fit to dive (certified by a physician according to German GTÜM guidelines). However, 72 dives were performed by divers with chronic diseases, 7 dives were done by divers smoking more than 1 pack*year, 60 by divers smoking less than 1 pack*year and 275 by non-smokers. Age and BMI distribution, as well as dive parameters, are displayed in Fig. 1 .

From a total of 342 dives, 161 dives were positive for visible bubbles in the right atrium and ventricle and also the inferior vena cava after the dive. Visible bubbles occurred especially in deep and long dives, divers over 30 years, and short surface intervals after the previous dive (residual inert gas supersaturation). Interrater reliability was 0.6 for all dives with main differences between EB grade 0 and 1 (2=6.3%, 3=3.3%, 4=1.2%, 5=0.3%). All disagreements were reevaluated in a third video rating. Relations of dive and individual parameters to bubble grades are displayed in Table 2 .

**Table:** **Table 2** Spearman’s correlation between echocardiographically detected bubble grades and individual as well as diving parameters.

Eftedal-Brubakk bubble grade 1–5	Spearman’s rho
Maximum Depth (meters)	0.46***
Air consumption equivalent to surface pressure (bar*l)	0.41***
Age of the diver (years)	0.25***
Dive time (minutes)	0.23***
Decompression dive	0.19***
Surface time before the dive (hours)	−0.12

(*** p<0.001,  ** p<0.01, *p<0.05, adjusted p-values). Surface time showed a significant linear correlation but p=0.05 only in Spearman’s categorical correlation with bubble grades.

No significant correlation was found between bubble grades and difference in blood pressure (p=0.056), heart rate (p=0.23) height (p=0.63), weight (p=0.84) and relative weight loss (p=0.19), freezing during the dive (categorical, p=0.14), impedance derived body muscle, fat and water contents (p>0.27), stress and problems during the dive (categorical, p>0.19), and smoking (categorical, p=0.54). Body weight adjusted pre-dive daily fluid intake showed a borderline correlation and minimal effect size only. Male divers were found to be diving deeper (t-test, p=0.002), longer (t-test, p=0.006), and consumed more compressed gas (t-test, p=0.000) than female divers, but there was no correlation of EB-Grade and gender (p=0.15).

### Distribution of correlated parameters within echocardiographic bubble grade categories

**Fig. 2:**
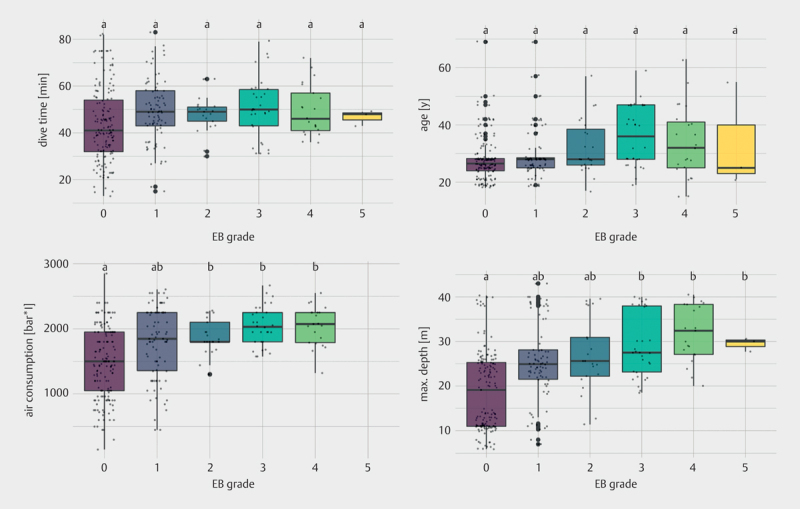
Eftedal-Brubakk bubble grades detected by echocardiography depending on depth, air consumption, age and surface time. Bubble grade 5 (whiteout) was visible after only 3 dives. Apart from fatigue in a few divers, that could be related with symptoms of a decompression incident but did not correlate with a higher bubble load, no other typical symptoms occurred. Small dots represent included data, and bold dots represent statistical outliers. Bars not sharing the same letters (a, b) are significantly different from each other (p<0.05, Tukey HSD test for unbalanced ANOVA), bars sharing the same letter are not significantly different from each other.

Since breathing while diving can only be done with a breathing gas pressure that is equal to the surrounding water pressure, the total air consumption in bar*l roughly combines the effects of dive time, depth and physical/psychological exertion – relevant for under water inert gas uptake – and is displayed in relation to the bubble grade detected via ultrasound in Fig. 2

### Multiple regression analysis of combined parameters and approximation of bubble grades

As shown in Table 1 , the EB scale does not resemble a linear scale of bubbles per cm ^2^ , thus the variable bubbles per cm ^2^ had to be transformed with a fourth root to achieve normal distribution of the residuals. Using multiple regression, a significant non-linear relationship between the response variable bubbles per cm ^2^ and the variables surface time, age, maximum depth, and air consumption was found ( Table 3 ).

**Table:** **Table 3** Results of multiple regression of the combined independent parameters.

Predictor	Standardized estimate (beta)	t-value (beta = 0)	p-value	VIF
Age (y)	0.216	3.960	0.000	1.02
Surface time (h)	−0.1433	−2.567	0.011	1.07
Max. depth (m)	0.308	4.207	0.000	1.83
Air consumption (bar*l)	0.177	2.477	0.014	1.76

The regression was calculated with the number of bubbles per cm ^2^ . The variance inflation factor (VIF<2) indicates no problematic multicollinearity within this model. The dependent variable was transformed beforehand with a double square root transformation. **R** ^**2**^ **=0.305** and adjusted R ^2^ =0.294 F(4, 239): 26.24, p-value <0.01.

To avoid bias of the regression model due to the fact that the experimental design is not fully cross-sectional and most individuals were surveyed for multiple dives, the individual factor was assessed using a random effects model, with the individual (ID) as random effect. For the random effects model with the same predictors as in the linear model we found a marginal pseudo-R ^2^ of 0.29 for the fixed effects and a conditional pseudo-R ^2^ of 0.37 for fixed and random effects, showing that the individual itself explained only a very small portion of the bubble grade (~8% of the variance, ICC (intra class correlation)=0.11). Moreover, all predictors later used in the simple regression were also significant in the random-effects regression (p<0.05 for age, surface time, max depth and air consumption) and therefore a simple linear approach was applicable. Furthermore, the data was subsampled multiple times into training and testing data (with a ratio of 30% and 70%), where the linear model proved to approximate the measured EB-grade correctly.

Thus, we can use the parameters as displayed in Table 4 to create a practical formula that predicts the bubble grade for an individual:

**Table:** **Table 4** Formula parameters to predict EB scale grades: R ^2^ =0.3131, Adjusted R ^2^ =0.3016; F(4,239): 27.23, p-value <0.001 (Constant=regression y-intercept).

Predictor	Estimate	Std. Error	p-value
Constant	−6.843*10 ^-01^	1.797*10 ^-01^	0.000
Age (y)	1.968*10 ^-02^	4.861*10 ^-03^	0.000
Surface time (h)	−6.831*10 ^-03^	2.494*10 ^-03^	0.007
Max. depth (m)	2.285*10 ^-02^	5.441*10 ^-03^	0.000
Air consumption (bar*l)	2.302*10 ^-04^	8.955*10 ^-05^	0.011

This can be used to derive a useful “field formula” as follows in order to predict Eftedal-Brubakk bubble grades after a dive from minimal dive and individual parameters:

EB bubble grade [0–5] = (0.0196785*age [y; 15−69] −0.0068313 *surface time [h; 1−48] +0.0228502*max depth [m; 3−43] +0.0002302*air consumption [bar*l; 150−2850]) ^***2***^

which can be approximated by:

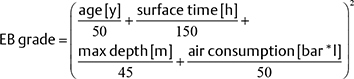



Equation 1: Field formula to approximate risk of bubbling.

**Fig. 3:**
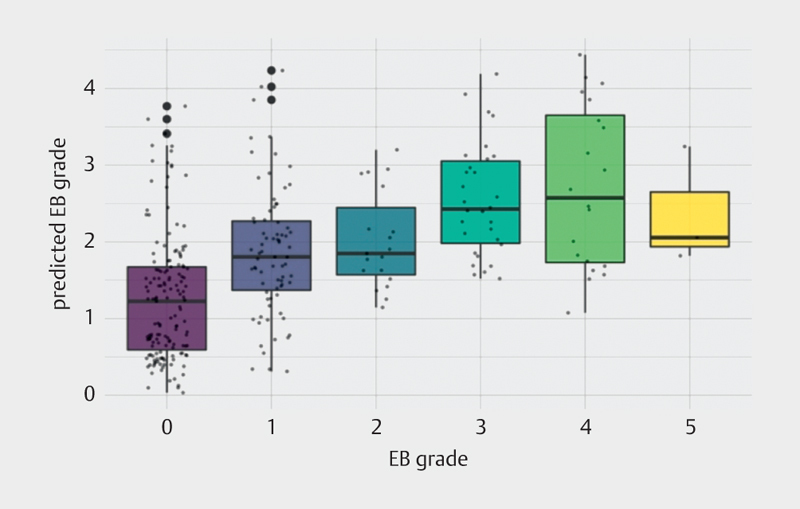
Prediction of Eftedal-Brubakk bubble grades using a formula of four variables only (age, surface time, maximum depth, and total air consumption). Small dots represent included data, and bold dots represent statistical outliers. The recording of surface time was limited to 48 h in our data, however, a surface time of more than 10 h did not reveal any difference.

Due to the categorical definition of the presence or absence of bubbles and the possible underestimation of grade 0, the intercept of −0.68 is not included into the formula ( Fig. 3 , Equation 1).

## Discussion

### Parameters correlating with bubble grade

In the past decades, there has been abundant research on safe diving parameters and decompression algorithms from an ex-ante view in order to avoid decompression stress and finally a decompression incident from critical inert gas supersaturation and major bubbling 14 . More recently, individual parameters causing a diving accident despite following empiric and calculated decompression rules were focused on from an ex-post view 15 16 . Today, ultrasound examinations after a dive, which are still done mainly by medical experts for research, add valuable information on individual decompression stress without symptoms of a diving accident 8 17 18 19 20 and help to initiate appropriate measures like extending surface intervals, breathing oxygen or increasing fluid intake to avoid a diving accident. Within a broad interindividual cohort and a variety of sports diving profiles within standard commercial dive computer limits, our study revealed bubbling in 47% of all dives and of all grades including whiteout. Known factors related to bubbling are diving exposure (depth, dive time, reduced surface interval), as well as BMI, age 7 and also diminished fluid status. Our study confirms the influence of diving exposure parameters. From individual factors, fat is known to increase inert gas storage capacity and is not related to higher bubble grades 16 right after the dive, as also confirmed by our data. Conflicting results 7 may be related to BMI-dependent impairment of physical condition and thus higher exertion and inert gas uptake. A repeated bubble grading was not carried out in this pilot study since precisely determined individual bubble peak curves were not relevant. The aim was instead to relate a broad spectrum of diving and individual parameters to post-dive bubbling at the same time interval of measurement that has already been found to be within the typical peak bubble time after sports SCUBA dives. The effect of small timely differences in bubble occurrence around the typical and previously published time interval of peak bubbling was expected to be lower than the effect of non-linear categorical bubble grading for a diver-oriented level of accuracy in detecting a relevant bubble load.

### Relevant parameters for bubble grade approximation

Most impressive is the strong correlation and moderate effect size of air consumption and age of the divers. Together with maximum depth and surface time, it was possible to find a formula predicting post-dive bubbling reasonably well. Although depth is related to air consumption of a dive, as is also dive time, depth still contributes a significant independent factor to bubble grade calculation more than dive time. This is due to the same air consumption that can occur in long shallow dives without relevant inert gas uptake compared to shorter deep dives with relevant inert gas saturation. Surface pressure equivalent air consumption in bar*l appeared to be a more suitable model mainly for diving exposure intensity and – to a smaller extent – individual metabolic activity than just maximum depth or diving duration. Some divers in our study seemed to be more prone to bubbling than others. However, after comparing dive profiles, we recognized a higher specific air consumption of these individuals. For example, male divers were diving deeper, longer, and consumed more air than female divers on average, yet there is no association with gender and EB grade in our data. All this suggests that the person-specific likelihood of an increased EB grade may be also a product of exposure intensity suitably shown by personal air consumption with further contributing individual factors such as age.

Several studies have shown that some individuals are more susceptible to DCI than others 15 21 22 . However, the individual effect was, in fact, measured in our model ICC (intra-class correlation coefficient)=0.11 and explained only about 8% of the variance of the EB grade (Pseudo-R ^2^ difference). The fixed effect parameters (depth, air consumption, surface time, age) explained the same variance in the model with the random effect (the individual) as in the model ignoring the random effect, we therefore proceeded with the latter, as the difference of this individual deviation to the estimated risk proved to be minor. The formula (Equation 1) can be seen as independent of this individual effect. Due to the high variability of the divers and diving profile a high variance was induced into our estimation, which can be expected in a field measurement.

### Limitations and potential for optimization and interpretation

We tried to find a simple, generalizable relation between dive and individual parameters in order to account for the additional information on relevant decompression stress, which can only be provided by a professional post-dive echocardiography. It was not possible for us to estimate whether individuals are susceptible differently to bubbling, as we did not have a standardized diving procedure and individual differences could also result from more risky diving behavior (i. e. there is a difference between men and women in the EB grade, but men also tend to dive deeper than women). With a standardized post-dive measurement, the individual and the dive parameters, as well as the resulting decompression stress, were our variable factors within the framework of standard sports diving profiles.

A shortcoming in our study is the categorical Eftedal-Brubakk scale of echo-bubble grading that we tried to accomplish with our non-linear formula. Especially in extreme bubble grades 0 and 5, the fit seems to be not optimal, yet the model can identify increased risks. Nevertheless, considering that a visible bubble grade 0 is challenging to judge, as a few bubbles that would define grade 1 or even grade 2 can be easily missed during approximately one minute of ultrasound scanning, the slight overestimation of our formula in this category seems to be quite realistic. On the other side of the scale, we had only three dives that showed a bubble grade 5 (whiteout) after the ascent. Furthermore, it is a big step between grade 4 (>1 bubble cm ^−2^ ) and whiteout without visible bubble discrimination. Therefore, it is challenging to predict grade 5 with our data reliably.

In order to find a better prediction of decompression stress with a possible linear relation, it seems necessary to leave the categorized scale towards a counted number of high-intensity transient signals, e. g., in ultrasound recordings of the inferior vena cava over time and semiautomatic counting 23 of visible or acoustic bubble signals. Further, timely variability of individual peak bubbling can be missed with our standardized, but single measurement approach. However, the relevant diagnostic information is not impaired by slight under- or overestimation of the bubble load. Other rough – more or less categorical – data like maximum depth (neglecting depth-time integral as much more fundamental factor for inert gas uptake), air consumption (neglecting air used for buoyancy control and the primary influence of depth) and EB bubble grading influence diagnostic accuracy significantly. Despite these biases, we were able to show a significant relation of a diver-oriented combination of simple individual and dive-related parameters to approximate relevant bubble load.

## Conclusion

It is possible to predict echocardiographically-derived bubble grading after a dive and therefore to generate information on decompression stress from inert gas bubbling in a categorical manner using a calculation based on easily accessible dive and individual parameters. Validation and adjustment with a large number of dives and a correlation to a more linear bubble grading with automatic integration, especially in dive computers with tank pressure sensors, could potentially contribute to individual diving safety.
